# Synthesis, Self-Assembly, and Cell Responses of Aromatic IKVAV Peptide Amphiphiles

**DOI:** 10.3390/molecules27134115

**Published:** 2022-06-27

**Authors:** Fang-Yi Wu, Hsin-Chieh Lin

**Affiliations:** 1 Department of Materials Science and Engineering, National Yang Ming Chiao Tung University, Hsinchu 300093, Taiwan, China; ivy10260527@gmail.com; 2 Department of Materials Science and Engineering, National Chiao Tung University, Hsinchu 300093, Taiwan, China

**Keywords:** self-assembly, peptide, amphiphile, hydrogel, biomaterial

## Abstract

Synthetic bioactive aromatic peptide amphiphiles have been recognized as key elements of emerging biomedical strategies due to their biocompatibility, design flexibility, and functionality. Inspired by natural proteins, we synthesized two supramolecular materials of phenyl-capped Ile-Lys-Val-Ala-Val (**Ben-IKVAV**) and perfluorophenyl-capped Ile-Lys-Val-Ala-Val (**PFB-IKVAV**). We employed UV-vis absorption, fluorescence, circular dichroism, and Fourier-transform infrared spectroscopy to examine the driving force in the self-assembly of the newly discovered materials. It was found that both compounds exhibited ordered π-π interactions and secondary structures, especially **PFB-IKVAV**. The cytotoxicity of human mesenchymal stem cells (hMSCs) and cell differentiation studies was also performed. In addition, the immunofluorescent staining for neuronal-specific markers of MAP2 was 4.6 times (neural induction medium in the presence of **PFB-IKVAV**) that of the neural induction medium (control) on day 7. From analyzing the expression of neuronal-specific markers in hMSCs, it can be concluded that **PFB-IKVAV** may be a potential supramolecular biomaterial for biomedical applications.

## 1. Introduction

Molecular self-assembly is the spontaneous behavior that makes molecules aggregate into well-defined sizes, shapes, and functions through noncovalent interactions [1,2,3,4,5]. It is speculated that simple and versatile molecular self-assembly systems can provide us with new perspectives to view some complex and unknown biological developments [6,7]. Among the self-assembled materials, self-assembling peptides have gained much attention owing to their biocompatibility, design flexibility, and functionality [8,9,10,11,12,13,14,15,16,17,18,19,20,21,22,23,24]. Self-assembling peptides with amphipathic structures are considered significant materials for building self-assembled nanostructures. The resulting nanostructures are highly bioactive and play a crucial role in materials science, regenerative medicine, tissue engineering, and drug delivery [25,26,27,28,29,30,31,32].

Aromatic peptide amphiphiles are currently the simplest and most effective method to develop low-molecular weight self-assembled biofunctional materials [33,34,35]. In aqueous solutions, aromatic peptide amphiphiles assemble by parallel, antiparallel, or interlocked antiparallel stacking arrangements to vesicles, micelles, nanotubes, and nanofibers, which subsequently cross-link into soft biomaterials [36,37,38,39,40]. For example, the 9-fluorenylmethoxycarbonyl (Fmoc) group was extensively used as an N-terminal aromatic component, and Fmoc-FF was a classic example of low molecular weight self-assembled biomaterials [36]. Aromatic incorporation with functional peptide sequences will afford more useful biomedical applications. It is known that laminin α1 chain-derived Ile-Lys-Val-Ala-Val (IKVAV) has diverse biological activities, including the promotion of cell adhesion, neural differentiation, and axon extension [41,42,43]. Many research results have reported improved neurite outgrowth of the stem cells with extracellular matrix scaffolds biofunctionalized with IKVAV motifs in the chemical structures of the materials. [44,45,46,47]. Roy et al. designed and synthesized Fmoc-IKVAV and Fmoc-YIGSR. They demonstrate the great potential of laminin-derived hydrogels in neuronal stem cell differentiation [48]. Thompson and Parish investigated Fmoc-DDIKVAV and successfully constructed strategies to improve stem cell therapy in brain repair [49].

We have recently developed a series of perfluorophenyl-capped peptides (PFB-peptides) and proved PFB is an effective N-terminal aromatic component to trigger the assembly of aromatic peptide amphiphiles [50,51,52,53,54]. In this research, we newly synthesized **Ben-IKVAV** (phenyl-capped pentapeptides) and **PFB-IKVAV**. We systematically investigate the self-assembly, microscopic morphology, mechanical, photophysical, and biological properties of **Ben-IKVAV** and **PFB-IKVAV**. It was found that **PFB-IKVAV** hydrogelator contains more β-sheet structures and has better cell differentiation ability than Ben-IKVAV, making it possible for tissue engineering and regenerative medicine application.

## 2. Results and Discussion

### 2.1. Molecular Design and Synthesis

**Figure 1:**
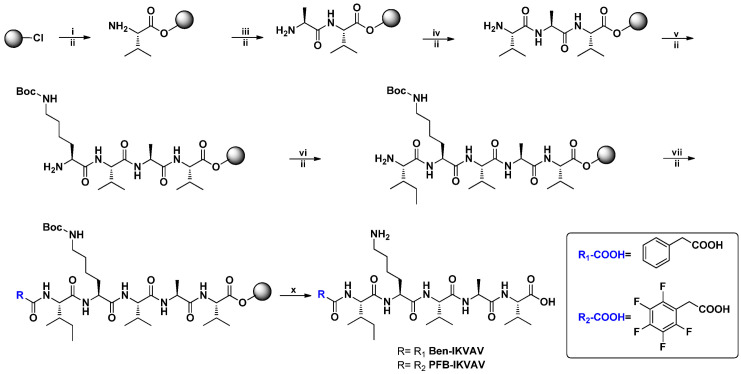
The synthetic route of **Ben-IKVAV** and **PFB-IKVAV**. (HBTU: *o*-Benzotriazol-1- yl-*N,N,N’,N’*-tetramethyluronium hexafluorophosphate; DIEA: *N,N*-diisopropylethyl- amine; TFA: trifluoroacetic acid.).

Self-assembling materials incorporating aromatic moieties with IKVAV peptides can be used as a platform technology to enhance the survival of human mesenchymal stem cells (hMSCs, 3A6) and their neuron differentiation. In this study, we modified Ben and PFB with the IKVAV peptide sequence for the novel materials of **Ben-IKVAV** and **PFB-IKVAV**. The synthetic route is shown in Figure 1; the peptide derivatives of **Ben-IKVAV** and **PFB-IKVAV** were prepared by the solid phase peptide synthesis (SPPS) method starting from 2-chlorotrityl chloride resin [55]. The peptide part was synthesized by adding Fmoc-_L_-Val-OH with coupling agents of HBTU and DIEA and then deprotection by piperidine. A similar procedure was repeated for Fmoc-_L_-Ala-OH, Fmoc-_L_-Val-OH, Fmoc-_L_-Lys(Boc)-OH and Fmoc-_L_-Ile-OH to grow the IKVAV peptide. Finally, 2-phenylacetic acid was added and treated with TFA to obtain **Ben-IKVAV**. The analog of **PFB-IKVAV** was afforded by replacing the 2-phenylacetic acid with 2-(perfluorophenyl)acetic acid. 

### 2.2. Investigation of Self-Assembly Properties

**Figure 2:**
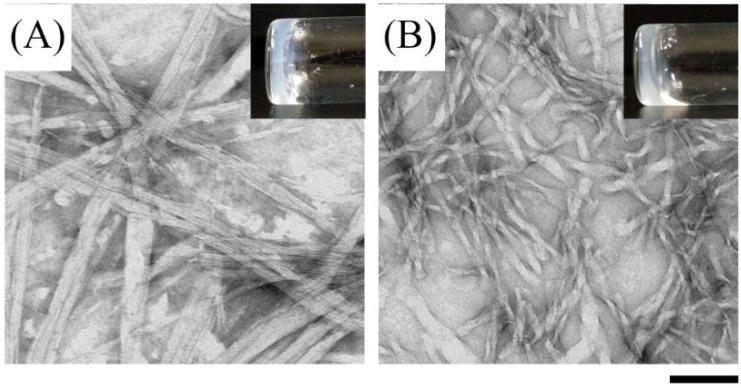
Optical (insets) and negatively stained TEM images of (**A**) **Ben-IKVAV** and (**B**) **PFB-IKVAV** at a concentration of 1 wt.% under pH 7.4. (Scale bar: 100 nm).

**Figure 3:**
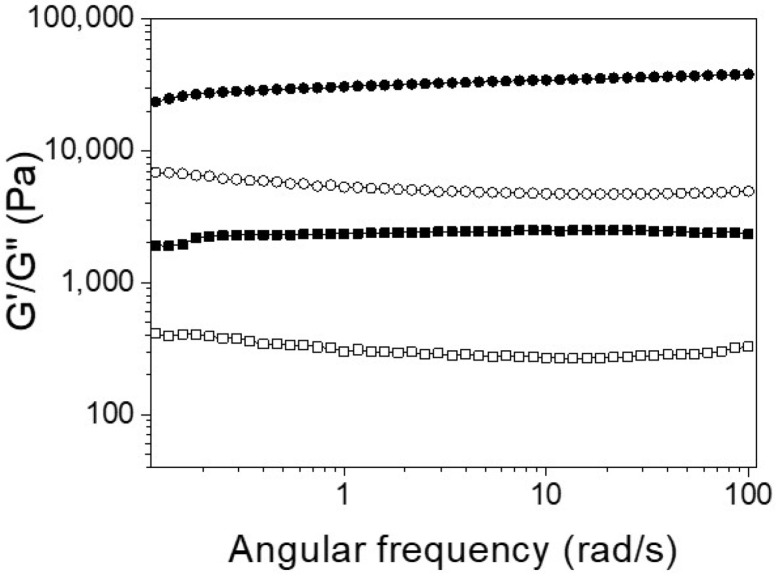
Frequency-dependent (ω = 0.1–100 rad·s^−1^) rheological measurement of **Ben-IKVAV** (circle) and **PFB-IKVAV** (square) at a concentration of 1 wt.% under pH 7.4 (solid for G′ and open for G′′).

For biomedical applications, the self-assembly properties of **Ben-IKVAV** and **PFB-IKVAV** were investigated under physiological conditions. It was found that both **Ben-IKVAV** and **PFB-IKVAV** (1 wt.%, ca. 15 mM) could form white translucent hydrogels under pH 7.4, and the optical images were shown in the insets of Figure 2. The corresponding microscopic morphologies were examined by transmission electron microscopy (TEM). As displayed in Figure 2, the negatively stained nanofiber structures of **Ben-IKVAV** were observed with a diameter of 4 ± 2 nm. The nanofibers are intertwined to form fiber bundles with a width of about 17 ± 1 nm. In contrast, **PFB-IKVAV** produces short nanofibers (diameter around 10 ± 1 nm). These fibrous nanostructures are distributed in water and entangled within a highly organized peptide-amphiphile nanofibers network (i.e., **Ben-IKVAV** and **PFB-IKVAV**), allowing for the formation of stable hydrogels. Moreover, the Tgel-sol of **Ben-IKVAV** and **PFB-IKVAV** were 65 and 55 °C, respectively. Figure 3 reveals the mechanical properties of 1 wt.% **Ben-IKVAV** and **PFB-IKVAV**, which were measured by oscillatory rheology. The storage modulus (G′) of **Ben-IKVAV** and **PFB-IKVAV** were 32.0 kPa and 2.4 kPa, respectively, suggesting they were suitable for applications in tissue engineering and regenerative medicine [56]. Furthermore, the self-assembly of **Ben-IKVAV** and **PFB-IKVAV** are viscoelastic gels, because the values of the G′ are higher than those of the loss modulus (G″) in both gels. 

### 2.3. Spectroscopic Characterization

**Figure 4:**
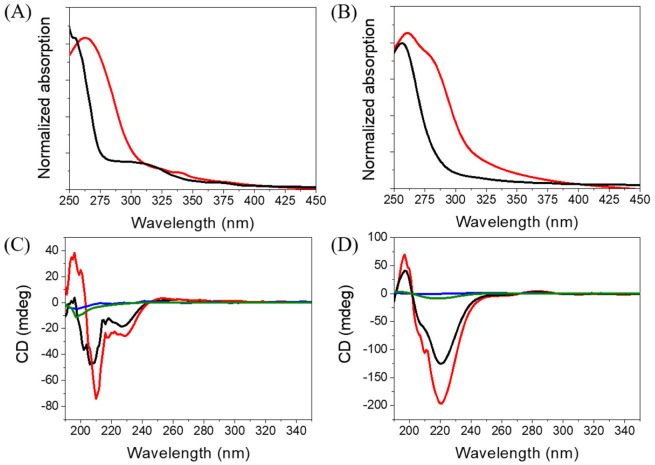
Absorption spectra of (**A**) **Ben-IKVAV** and (**B**) **PFB-IKVAV** at a concentration of 1 wt.% (red for water and black for methanol). CD spectra of (**C**) **Ben-IKVAV** and (**D**) **PFB-IKVAV** in water (blue for 50 μM, green for 500 μM, black for 1000 μM, and red for 5000 μM).

**Figure 5:**
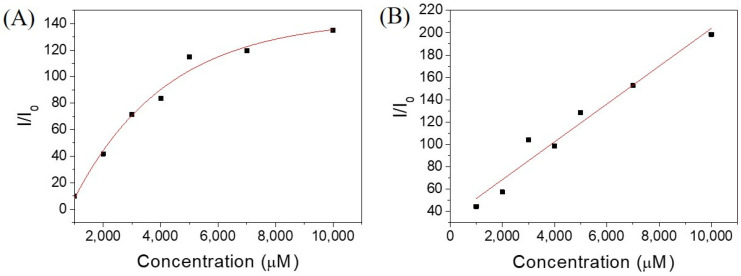
Emission intensities of ThT at various concentrations of hydrogelators of (**A**) **Ben-IKVAV** and (**B**) **PFB-IKVAV**. (I_0_: without adding hydrogelators.).

Since **Ben-IKVAV** and **PFB-IKVAV** could self-assemble to form the network of nanofibers and afford the hydrogels, we further studied the driving force for the intermolecular interactions of the hydrogelators using spectroscopic characterization [57,58]. We found that the compounds uniformly dispersed in methanol with disordered ar-rangements, which is different from that in water. The UV-vis absorption and circular dichroism (CD) spectra of **Ben-IKVAV** and **PFB-IKVAV** in water and methanol are presented in Figure 4. The absorption peaks of **Ben-IKVAV** are at 262 nm and 254 nm in water and methanol, respectively, indicating the redshift feature caused by the aggregation formation in an aqueous solution. Interestingly, for **PFB-IKVAV**, except for the redshift of the main absorption peak in water, a new absorption band around 285 nm appeared, presumably attributed to the denser aggregation of the PFB units in **PFB-IKVAV**. CD spectra were recorded to understand the structural properties and molecular arrangement of **Ben-IKVAV** and **PFB-IKVAV** (Figure 4C,D). At low concentrations of **Ben-IKVAV** and **PFB-IKVAV** (50 and 500 μM) in water, no CD signals were detected in the wavelength region 190–350 nm. Increasing the concentration to 1000 μM, both compounds exhibited a significant Cotton effect in the aromatic ring region, revealing they may have π-π interactions between molecules and accompany an orderly arrangement in the aqueous environment. Meanwhile, we also observed that the hydrogelators of **Ben-IKVAV** and **PFB-IKVAV** had a negative peak at 210–220 nm and a positive peak around 197 nm, indicating that they have β-sheet conformation in the self-assembly pentapeptide hydrogelators [59]. These results suggest that **Ben-IKVAV** and **PFB-IKVAV** may self-assemble into the high-order supramolecular structures through π-π interactions and hydrogen bonding when the concentration is higher than 1000 μM. In addition, Fourier-transform infrared spectroscopy (FT-IR) was employed to verify the hydrogen interactions between molecules in the self-assembled state. As revealed by FT-IR, the FT-IR spectra of **Ben-IKVAV** and **PFB-IKVAV** at 5000 μM in water displayed strong absorption peaks at 1630 cm^−1^, demonstrating the hydrogen bonding interaction in the assemblies (Figure S1, Supplementary Materials) [60]. Since thioflavin T (ThT) is a well-known fluorescent dye utilized to identify the presence of amyloid fibrils [61], we used the ThT fluorescence assay to further confirm the fibrillar peptide structures present in **Ben-IKVAV** and **PFB-IKVAV**. It is known that when ThT binds to β-amyloid fibrils, a remarkable enhancement in fluorescence intensity can be detected at 484 nm. Figure 5 shows the ratio of the emission intensities of ThT at various concentrations of **Ben-IKVAV** and **PFB-IKVAV**. It was found that the calculated fluorescence enhancement ratios were 140 and 210, respectively, suggesting that the self-assembly of **Ben-IKVAV** and **PFB-IKVAV** may exhibit β-sheet-like conformation and more folded secondary structures in the presence of **PFB-IKVAV**. Notably, **PFB-IKVAV** has a larger electronegativity than **Ben-IKVAV** at the terminal end of the molecule, thereby having different hydrogen-bonding interactions and resulting in the different behavior of fluorescence intensity with ThT.

### 2.4. Cytotoxicity Test

**Figure 6:**
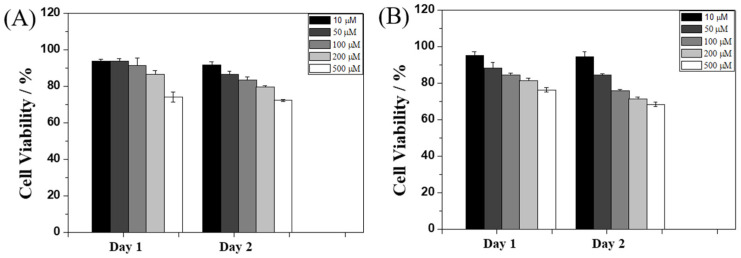
Viability ratio measured using MTT assay on hMSCs in the presence of 10, 50, 100, 200, and 500 μM of (**A**) **Ben-IKVAV** and (**B**) **PFB-IKVAV** for 48 h.

To evaluate the potential biological applications of **Ben-IKVAV** and **PFB-IKVAV**, we performed a biocompatibility test of these hydrogelators against hMSCs through MTT [3-(4,5-dimethylthiazol-2-yl)-2,5-diphenyltetrazolium bromide] assay [62], and the results are presented in Figure 6. The concentrations of **Ben-IKVAV** and **PFB-IKVAV** were selected in the range of 10–500 μM, and the cell viability measurements were processed at 24 and 48 h after cell seeding. We found that the half-inhibitory concentration (IC_50_) of the two compounds was greater than 500 μM, and the cell viabilities were over 75% when cultured at a concentration of 500 μM for 48 h. Therefore, it can be proved that the two compounds have good biocompatibility and can be further used for cell differentiation studies.

### 2.5. Cell Differentiation Study

**Figure 7:**
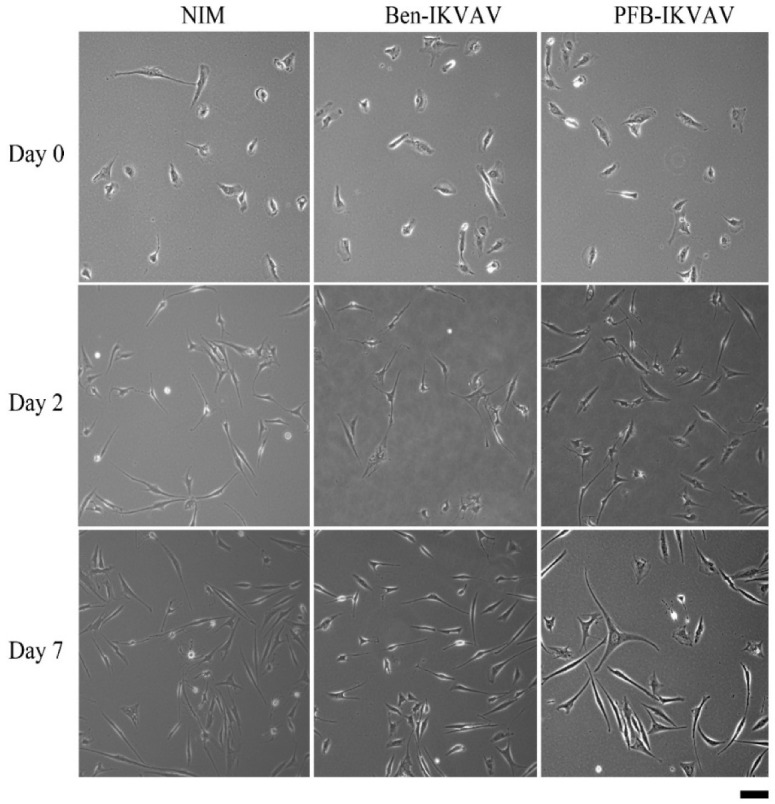
The optical microscope images of cell morphology of hMSCs cultured with NIM (control, left), NIM in the presence of **Ben-IKVAV** (middle)**,** and NIM in the presence of **PFB-IKVAV** (right) for 2 days and 7 days. (Scale bar: 50 μm).

**Figure 8:**
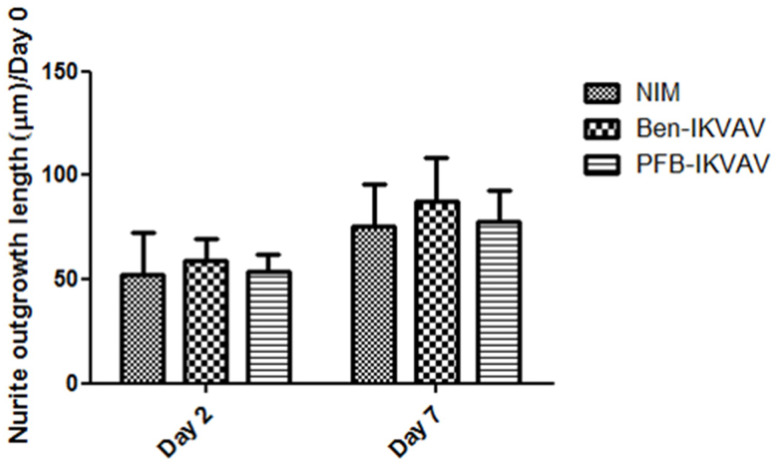
Neurite outgrowth lengths of hMSCs cultured with NIM (control), **Ben-IKVAV** in NIM, and **PFB-IKVAV** in NIM for 2 days and 7 days (*n* = 50).

Since **Ben-IKVAV** and **PFB-IKVAV** are biocompatible, we then diluted 1 wt.% of **Ben-IKVAV** and **PFB-IKVAV** hydrogels with basal medium and neural induction medium (NIM) to 1000 μM, respectively, and cultured them with hMSCs for 0, 2, and 7 days (Figure S2 and Figure 7). From the experimental results, hMSCs cultured with NIM, in the presence of **Ben-IKVAV** or **PFB-IKVAV,** could successfully differentiate hMSCs into neuron-like cells (Figure 7). The fibrous structures of **Ben-IKVAV** or **PFB-IKVAV** scattered in the culture medium (NIM) are combined with cell surface receptors to promote neural differentiation. To understand the effect of neural differentiation of hMSCs, the average neurite outgrowth lengths on days 2 and 7 were measured in the control and experimental groups (i.e., **Ben-IKVAV** in NIM and **PFB-IKVAV** in NIM). As shown in Figure 8, there was no significant difference in cell morphology and axon extension length; we further investigated the molecular biology analysis of the three samples to distinguish the effect of the materials.

**Figure 9:**
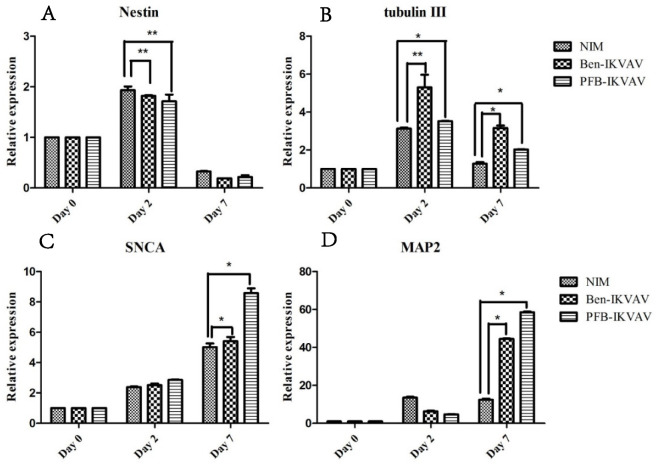
RT-qPCR analysis of neuronal-specific markers (**A**) Nestin, (**B**) β-tubulin, (**C**) SNCA, and (**D**) MAP2 of hMSCs cultured with NIM (control), NIM in the presence of **Ben-IKVAV,** and NIM in the presence of **PFB-IKVAV** for 2 days and 7 days (*: *p* < 0.05, **: *p* < 0.01).

To investigate whether IKVAV derivatives can effectively promote the differentiation of stem cells into neural cells, we diluted 1 wt.% of **Ben-IKVAV** and **PFB-IKVAV** hydrogels with NIM to 1000 µM, respectively, and cultured hMSCs for 0, 2, and 7 days. The mRNA was collected from NIM, NIM in the presence of **Ben-IKVAV**, and NIM in the presence of **PFB-IKVAV** and subjected to real-time quantitative polymerase chain reaction (RT-qPCR) analysis [63]. The expression of neuronal-specific markers, Nestin, β-tubulin, SNCA, and MAP2 are analyzed in this work [64,65,66,67]. As can be seen from Figure 9, it was found that the expression levels of Nestin and β-tubulin increased on day 2. However, the expression levels of both gradually decreased with the increase in differentiation days (i.e., day 7). The marker genes for the middle and late stages of neurons were all up-regulated, especially the expression level of MAP2 genes was significantly increased on day 7. Although we did not observe a noticeable difference in cell morphology between NIM (control), NIM in the presence of **Ben-IKVAV,** and NIM in the presence of **PFB-IKVAV** in Figure 7, there were significant differences in the expression of nerve cell-specific marker genes (Figure 9). These results suggest that **Ben-IKVAV** and **PFB-IKVAV** may have begun to regulate the differentiation mechanism in the cells and can effectively promote the trend of mesenchymal stem cells towards neural cell differentiation. Additionally, **PFB-IKVAV** has shown a better cell differentiation ability than **Ben-IKVAV**.

**Figure 10:**
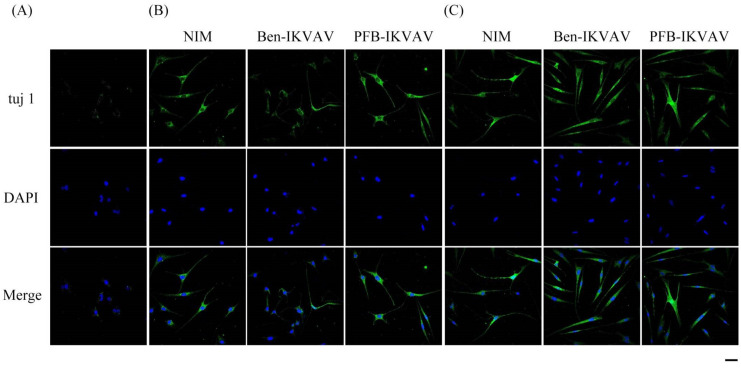
Representative images showing immunofluorescent staining of β-tubulin (tuj 1) expression in hMSCs cultured with NIM (control), NIM in the presence of **Ben-IKVAV,** and NIM in the presence of **PFB-IKVAV** for (**A**) day 1, (**B**) day 2, and (**C**) day 7. Upper: cells appear green in color due to the presence of β-tubulin-Ab; middle: nuclei appear blue due to staining with DAPI; lower: merged image. (Scale bar: 20 μm.).

**Figure 11:**
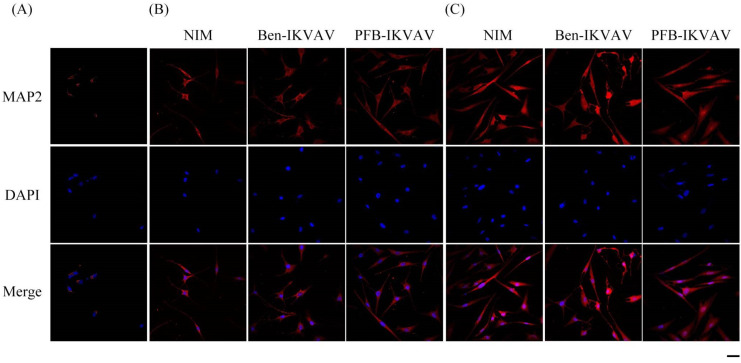
Representative images show immunofluorescent staining of MAP2 expression in hMSCs cultured with NIM (control), NIM in the presence of **Ben-IKVAV,** and NIM in the presence of **PFB-IKVAV** for (**A**) day 1, (**B**) day 2, and (**C**) day 7. Upper: cells appear red due to the presence of MAP2-Ab; middle: nuclei appear blue due to staining with DAPI; lower: merged image. (Scale bar: 20 μm).

After neuronal inductions of hMSCs, immunofluorescent staining for neuronal-specific markers of β-tubulin and MAP2 was achieved [68,69]. Figure 10 presents immunostaining for β-tubulin (green) with DAPI (blue) co-staining in hMSCs cultured with NIM (control), NIM in the presence of **Ben-IKVAV,** and NIM in the presence of **PFB-IKVAV** for days 1, 2, and 7. It can be seen that there is no β-tubulin stained on day 1. As the culture time increased to day 2, the expression of β-tubulin in the control group and the experimental groups showed an increasing trend, indicating a trend of neural differentiation and the extension of neural axons. On the 7th day of culture, a more evident increase of β-tubulin was observed. Immunostaining for MAP2 (red) with DAPI (blue) co-staining showed the similarity results (Figure 11); more interestingly, in addition to the more pronounced increase in MAP2, the expression of MAP2 was also found in the nucleus on day 7. It is speculated that the nuclear entry may be related to the initiation of the regulation of the gene level in the nucleus to make the differentiation more complete. An analysis of neuronal-specific markers by immunostaining revealed that the morphology of neuronal cells changes were sustained with the expression of β-tubulin and MAP2 in hMSCs (Figure 10 and Figure 11). According to our observation, the cell morphology changed significantly after 2 days of culture in NIM, in the presence of **Ben-IKVAV** and **PFB-IKVAV**. After 7 days of differentiation, the cells gradually tended to mature nerve cells. Among these materials, **PFB-IKVAV** is the best one for helping cell differentiation.

## 3. Materials and Methods

### 3.1. Materials and Apparatus

Chemicals were obtained from Aldrich unless specified otherwise. ^1^H NMR (nuclear magnetic resonance spectroscopy) spectra were measured in DMSO-*d_6_* using a 300 MHz NMR spectrometer. The optical microscope images of cell morphology were recorded with a CLSM (Leica TCS SP5X, Wetzlar, Germany).

### 3.2. Synthesis of Ben-IKVAV 

2-Chlorotrityl chloride resin (1.2 g, 1.000 mmol) was swelled in anhydrous CH_2_Cl_2_ for 30 min, and then Fmoc-_L_-Val-OH (0.678 g, 2.000 mmol) was loaded onto the resin in anhydrous DIEA (0.830 mL, 5.000 mmol) for 1 h. For deprotection of the Fmoc group, 20% piperidine was added, and the sample was left for 30 min; this procedure was repeated twice (each time for 2 min). Fmoc-_L_-Ala-OH (0.851 g, 2.000 mmol) was reacted to the amino group using HBTU (0.758 g, 2.000 mmol) and DIEA (0.830 mL, 5.000 mmol) as coupling agents for 30 min. Again, the sample was treated with 20% piperidine for 30 min; this procedure was repeated twice (each time for 2 min). Fmoc-_L_-Val-OH (0.678 g, 2.000 mmol) was coupled to the amino group using HBTU (0.758 g, 2.000 mmol) and DIEA (0.830 mL, 5.000 mmol) as coupling agents for 30 min. Again, the sample was treated with 20% piperidine for 30 min; this procedure was repeated twice (each time for 2 min). Fmoc-_L_-Lys(Boc)-OH (0.937 g, 2.000 mmol) was coupled to the amino group using HBTU (0.758 g, 2.000 mmol) and DIEA (0.830 mL, 5.000 mmol) as coupling agents for 30 min. Again, the sample was treated with 20% piperidine for 30 min; this procedure was repeated twice (each time for 2 min). Fmoc-_L_-Ile-OH (0.707 g, 2.000 mmol) was coupled to the amino group using HBTU (0.758 g, 2.000 mmol) and DIEA (0.830 mL, 5.000 mmol) as coupling agents for 30 min. Again, the sample was treated with 20% piperidine for 30 min; this procedure was repeated twice (each time for 2 min). Finally, 2-phenylacetic acid (0.372 g, 3.000 mmol) was coupled to the free amino group using HBTU (1.138 g, 3.000 mmol) and DIEA (1.250 mL, 7.500 mmol) as coupling agents. After the reaction mixture was stirred overnight, the peptide derivative was cleaved through treatment with TFA. The resulting solution was dried under air, and then DI water was added to precipitate the target product. The solid was dried under a vacuum to remove the residual solvent (light brown solid: 0.467 g) (Figures S3 and S5). ^1^H NMR (300 MHz, DMSO-*d_6_*): δ = 0.70–1.00 (m, 18H, CH_3_), 1.00–1.05 (m, 1H, CH_2_), 1.05–1.35 (m, 2H, CH_2_), 1.40–1.60 (m, 4H, CH_2_), 1.65–1.70 (m, 2H, CH_2_), 1.95–2.15 (m, 2H, CH_2_), 2.70–3.20 (m, 4H, CH_2_), 4.10–4.50 (m, 5H, CH), 7.20–7.40 (m, 5H, CH), 7.60–7.75 (m, 3H, NH), 7.91 (d, *J* = 9.1 Hz, 1H, NH), 8.07 (d, *J* = 7.2 Hz, 1H, NH), 8.10–8.25 (m, 2H, NH); ^13^C NMR (75 MHz, DMSO-*d_6_*): δ = 10.9, 15.4, 17.9, 18.2, 19.06, 19.15, 22.3, 24.3, 26.6, 30.0, 30.7, 31.1, 36.6, 42.1, 48.0, 52.4, 56.9, 57.1, 57.3, 126.3, 128.2, 129.0, 136.7, 170.2, 170.4, 171.2, 171.3, 172.3, 172.8. MS [ESI^+^]: calcd. *m/z* 646.82, obsvd. 647.52 [M−H]^+^. 

### 3.3. Synthesis of PFB-IKVAV

A similar procedure was used for Fmoc-_L_-Val-OH, Fmoc-_L_-Ala-OH, Fmoc-_L_-Val-OH, Fmoc-_L_-Lys(Boc)-OH, and Fmoc-_L_-Ile-OH to grow the IKVAV peptide. Subsequently, 2-(perfluorophenyl)acetic acid (0.678 g, 3.000 mmol) was coupled to the free amino group using HBTU (1.138 g, 3.000 mmol) and DIEA (1.250 mL, 7.500 mmol) as coupling agents. After the reaction mixture had been stirred overnight, the aromatic-capped peptide derivative was cleaved through treatment with TFA. The resulting solution was dried by air, and then DI water was added to precipitate the target product. The solid material was dried under a vacuum to remove the residual solvent (light brown solid: 0. 592 g) (Figures S4 and S6). ^1^H NMR (300 MHz, DMSO-*d_6_*): δ = 0.75–1.00 (m, 18H, CH_3_), 1.05–1.15 (m, 1H, CH_2_), 1.15–1.30 (m, 4H, CH_2_), 1.30–1.40 (m, 2H, CH_2_), 1.45–1.60 (m, 4H, CH_2_), 1.60–1.85 (m, 2H, CH_2_), 1.95–2.15 (br, 2H, CH_2_), 3.65–3.85 (m, 2H, CH_2_), 4.10–4.50 (m, 5H, CH), 7.65–7.80 (m, 3H, CH), 7.91 (d, *J* = 8.1 Hz, 1H, NH), 8.06 (d, *J* = 6.9 Hz, 1H, NH), 8.19 (d, *J* = 8.1 Hz, 1H, NH), 8.42 (d, *J* = 9.1 Hz, 1H, NH); ^13^C NMR (75 MHz, DMSO-*d_6_*): δ = 12.0, 16.3, 18.8, 19.1, 20.0, 20.1, 23.3, 25.2, 27.6, 29.6, 30.9, 31.7, 32.1, 37.8, 48.9, 53.5, 58.1, 58.3, 117.8, 137.8, 145.9, 167.8, 171.3, 171.8, 172.3, 173.3, 173.8. MS [ESI^+^]: calcd. *m*/*z* 736.77, obsvd. 737.47 [M−H]^+^.

### 3.4. Cell Viability Tests 

The biocompatibilities of **Ben-IKVAV** and **PFB-IKVAV** were measured by the MTT cell viability test. The hMSCs were seeded in 24-well plates (density: 50,000 cells per well) with Dulbecco’s modified Eagle’s medium (DMEM, 0.5 mL) containing 10% phosphate-buffered saline (PBS, pH 7.4) and 1% penicillin/streptomycin solution and then incubated for 24 h. Compounds of **Ben-IKVAV** and **PFB-IKVAV** at different concentrations (10, 50, 100, 200, and 500 µM) were added when cells were plated. After 24 and 48 h, the medium was replaced with fresh medium supplemented with MTT reagent (4 mg mL^–1^, 0.5 mL per well). After another 4 h, the medium containing MTT was removed, and DMSO (0.5 mL per well) was added to dissolve the formazan crystals. Each of the 24 wells was transferred to a 96-well plate. The optical densities (ODs) of the resulting solutions were measured at 595 nm using an absorbance microplate reader (Infinite F50, TECAN, Männedorf, Switzerland). Cells that had not been subjected to treatment with the compounds were used as the control. The cell viability percentage (%) was calculated from the expression OD_sample_/OD_control_.

### 3.5. Cell Differentiation

The hMSCs were grown in 24-well plates (density: 2.4 × 10^5^ cells per well) in NIM supplemented with 1% penicillin/streptomycin, 10^−7^ M dexamethasone, 50 μg/mL _L_-Ascorbic acid-2 phosphate, 50 μM indomethacin, 10 μg/mL insulin, and 0.45 mM 3-isobutyl-1-methyl-xanthine. The cells cultured in a basal medium were used as a negative control.

### 3.6. Real-Time Quantitative Polymerase Chain Reaction

Total RNA was isolated from the control and induced (Neurogenic) hMSCs and quantified with a spectrophotometer (NanoPhotometer™ Pearl Design Edition, Westlake Village, CA, USA). Complementary DNA (cDNA) was synthesized from the total purified RNA (1 μg) using a MMLV High-Performance Reverse Transcriptase kit with a 10 μM OligodT primer at 37 °C for 1 h. The RT-qPCR reaction was achieved with cDNA quantified with 2 × SYBR Green supermix supplemented with 10 μM of specific primers set (Table 1). The RT-qPCR reaction was carried out with initial denaturation at 95 °C for 10 min, followed by 40 cycles of PCR at 95 °C for 15 s, and 60 °C for 60 s.

### 3.7. Immunofluorescent Staining

After neuronal inductions of hMSCs, immunostaining was performed for neuronal-specific markers. Cells were fixed with 4% formaldehyde for 15 min and permeabilized with PBST buffer (0.1% Triton X-100 supplemented with BSA). After permeabilization, cells were blocked with Blocking buffer (PBST buffer + 5% serum) for 30 min and then incubated with primary antibodies β-tubulin (1:100) and MAP2 (1:200) at 4 °C overnight. After primary antibody treatment, cells were washed with PBST buffer and incubated with secondary antibodies, FITC conjugated goat-anti-mouse (β-tubulin), and cy5 conjugated goat-anti-rabbit (MAP2), for 1 h. For nuclear staining, cells were treated with 4′,6-diamidino-2-phenylindole (DAPI) for 15 min. Finally, cells were observed under a fluorescence microscope.

## 4. Conclusions

In summary, we have designed and synthesized newly aromatic peptide amphiphiles of **Ben-IKVAV** and **PFB-IKVAV**. We systematically investigated the self-assembly, microscopic morphology, mechanical, and photophysical properties by using transmission electron microscopy, rheology, UV-vis absorption, fluorescence, circular dichroism, and Fourier-transform infrared spectroscopy. It was found that both compounds displayed order π-π interactions and β-sheet structures in the assemblies, especially **PFB-IKVAV**. Our findings demonstrate the importance of the amphiphilic molecular design of self-assembled supramolecular nanomaterials. The cytotoxicity of human mesenchymal stem cells and cell differentiation studies was also performed, indicating that **PFB-IKVAV** is a potential biomaterial opening new perspectives for future investigations into tissue engineering and regenerative medicine.

## Figures and Tables

**Table 1:** List of primers used in RT-qPCR.

Gene	Primer Sequence	Product Size (bp)
Nestin	5′-CTGGAGCAGGAGAAACAGG-3′ (forward) 5′-TGAAAGCTGAGGGAAGTCTTG-3′ (reverse)	182
β-tubulin	5′-AGCAAGAACAGCAGCTACTTCGT -3′ (forward) 5′-GATGAAGGTGGAGGACATCTTGA -3′ (reverse)	102
α-synuclein (SNCA)	5′-AGGACTTTCAAAGGCCAAGG-3′ (forward) 5′-TCC TCCAACATTTGTCACTTG-3′ (reverse)	187
MAP2	5′-CTTCAGCTTGTCTCTAACCGAG-3′ (forward) 5′-CCTTTGCTTCATCTTTCCGTTC-3′ (reverse)	199

## Data Availability

Not applicable.

## Supplementary Materials

The following supporting information can be downloaded at: https://www.mdpi.com/article/10.3390/molecules27134115/s1, Figure S1: FT-IR spectra of (A) **Ben-IKVAV** and (B) **PFB-IKVAV** at a concentration of 5000 μM in water; Figure S2: The optical microscope images of cell morphology of hMSCs cultured with basal medium (control), basal medium in the presence of **Ben-IKVAV**, and basal medium in the presence of **PFB-IKVAV** for 2 days and 7 days (scale bar: 50 μm); Figure S3: ^1^H NMR spectrum of **Ben-IKVAV** in DMSO-*d_6_*; Figure S4: ^1^H NMR spectrum of **PFB-IKVAV** in DMSO-*d_6_*; Figure S5: ^13^C NMR spectrum of **Ben-IKVAV** in DMSO-*d_6_*; Figure S6: ^13^C NMR spectrum of **PFB-IKVAV** in DMSO-*d_6_*.
