# Supplementary material for: Synthesis, Self-Assembly, and Cell Responses of Aromatic IKVAV Peptide Amphiphiles
Source: Molecules. 2022 Jun 27;27(13):4115. doi: 10.3390/molecules27134115 (PMC9267992; doi:10.3390/molecules27134115)

Supplementary Information (SI)

Figure S1. FT-IR spectra of (A) **Ben-IKVAV** and (B) **PFB-IKVAV** at a concentration of 5000 μM in water.

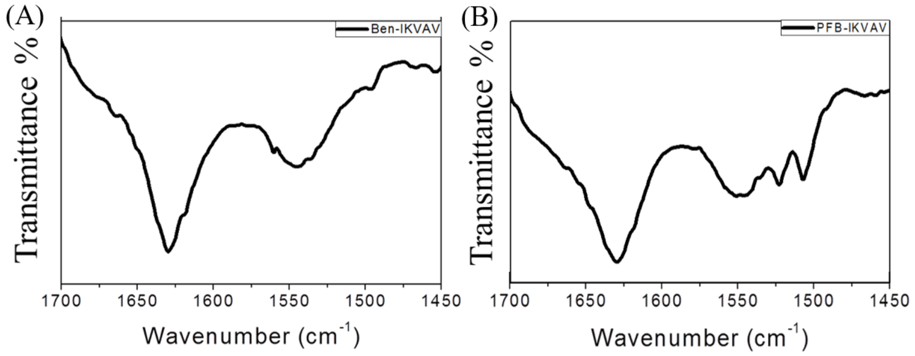

Figure S2. The optical microscope images of cell morphology of hMSCs cultured with basal medium (control), basal medium in the presence of **Ben-IKVAV**, and basal medium in the presence of **PFB-IKVAV** for 2 days and 7 days. (Scale bar: 50 μm)

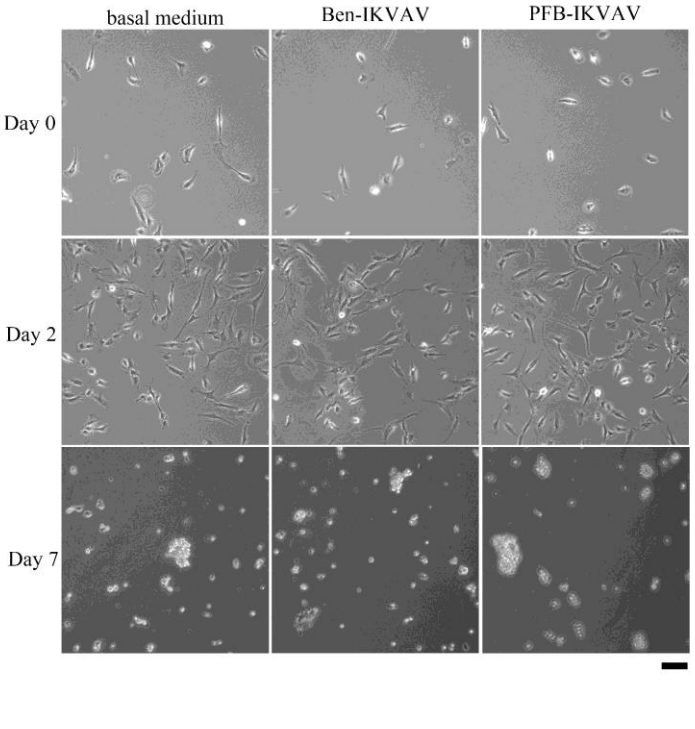

Figure S3. ^{1}H NMR spectrum of **Ben-IKVAV** in DMSO-d_6.

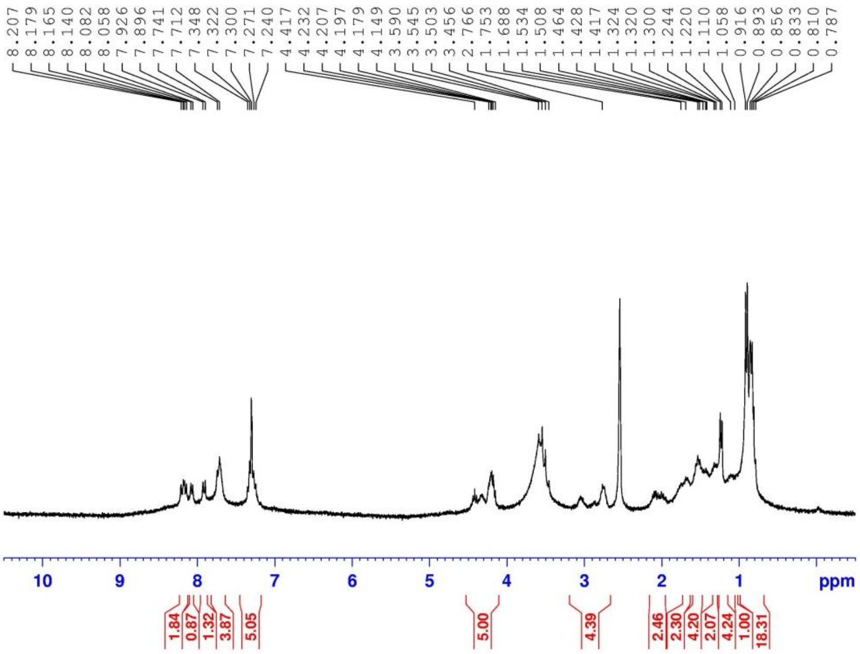

Figure S4. ^{1}H NMR spectrum of **PFB-IKVAV** in DMSO-*d6*.

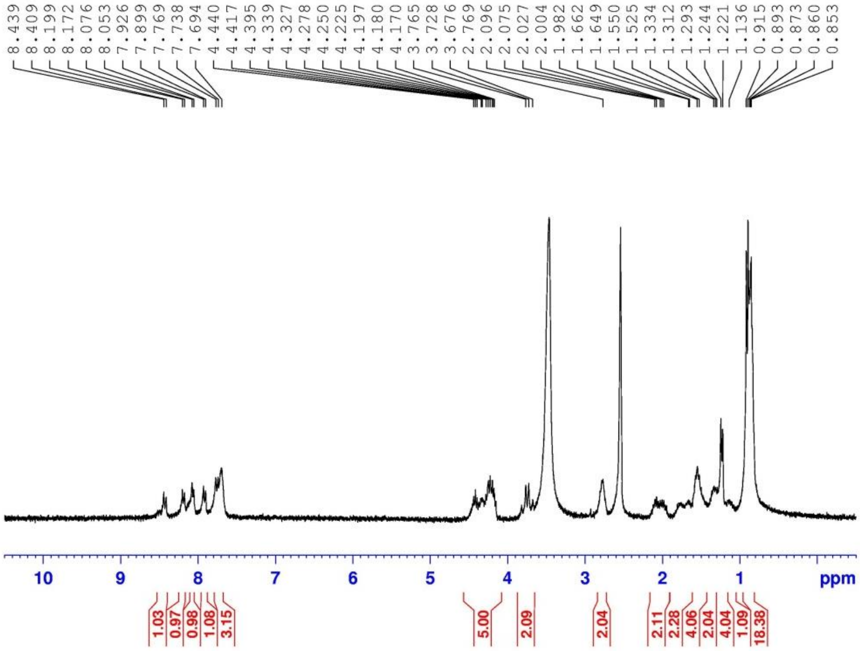

Figure S5. ^{13}C NMR spectrum of **Ben-IKVAV** in DMSO-*d6*.

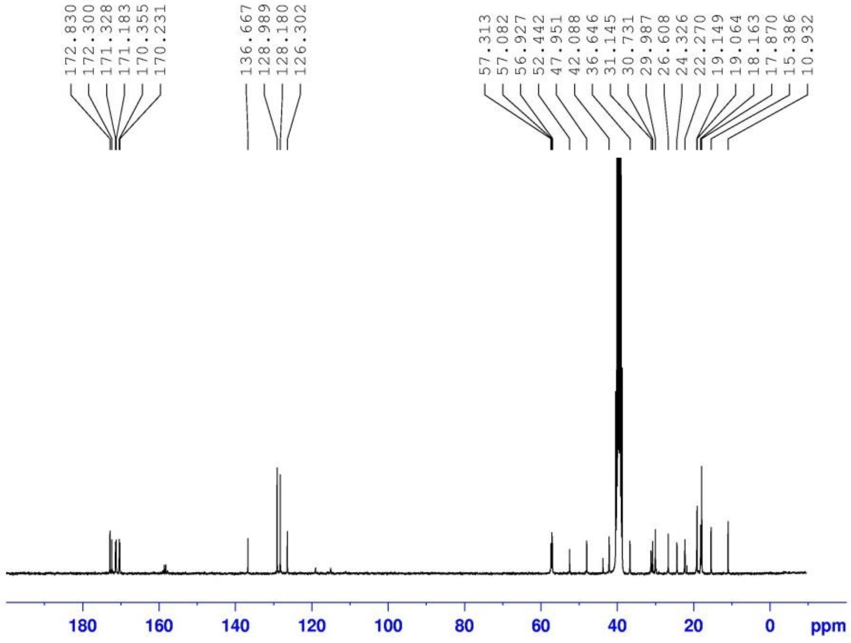

Figure S6. ^{13}C NMR spectrum of **PFB-IKVAV** in DMSO-d_6.

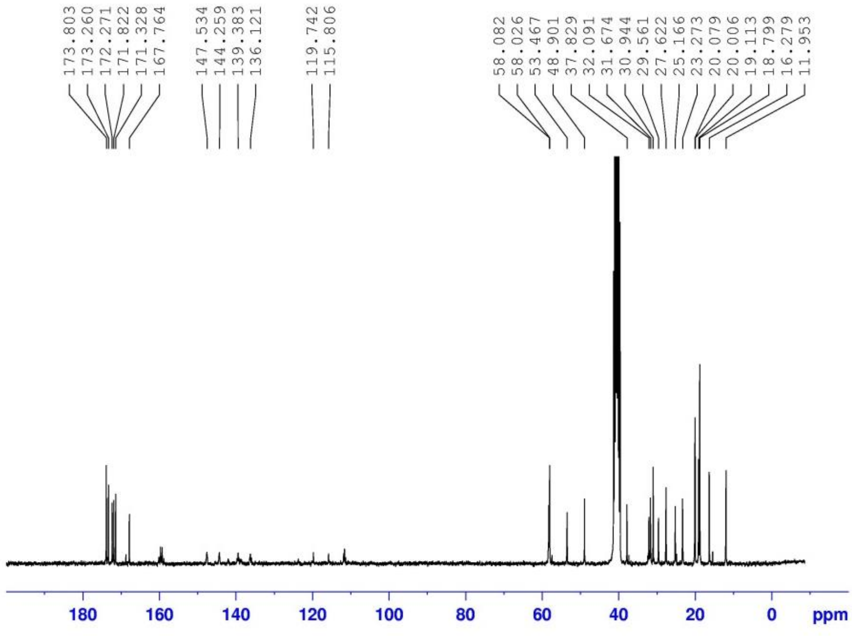

Supplement: Supplementary file 1 [file molecules-27-04115-s001.zip › molecules-1710426-supplementary.pdf]
